# All-Cause Hospitalisation After Endoscopic Diagnosis of Diverticular Disease: A Prospective Real-World Cohort Study

**DOI:** 10.3390/jcm15166385

**Published:** 2026-08-18

**Authors:** Anna Maria Pietrzak, Agnieszka Chreptowicz, Marcin Stanisław Pietrzak, Andrzej Mróz

**Affiliations:** 1 2nd Department of Gastroenterology, Centre of Postgraduate Medical Education, Marymoncka 99/103 Str., 01-813 Warsaw, Poland; 2 Department of Gastroenterology, Bielanski Hospital, Ceglowska 80 Str., 01-809 Warsaw, Poland; 3 Endoscopy Department, Ministry of the Interior and Administration Policlinic, Szpitalna 2 Str., 75-720 Koszalin, Poland; chrepcia82@wp.pl; 4 Faculty of Medicine, Medical University of Warsaw, 61 Zwirki i Wigury Str., 02-091 Warsaw, Poland; 5 Department of Pathology, Centre of Postgraduate Medical Education, Marymoncka 99/103 Str., 01-813 Warsaw, Poland; andrewcio@wp.pl; 6 Department of Pathology, Bielanski Hospital, Ceglowska 80 Str., 01-809 Warsaw, Poland

**Keywords:** diverticular disease, diverticulitis, DICA scale, healthcare utilisation, multimorbidity, real-world evidence

## Abstract

**Background**: Diverticular disease (DD) represents an important clinical and healthcare burden, particularly in ageing populations. The relationship between the Diverticular Inflammation and Complication Assessment (DICA) classification and overall healthcare utilisation remains insufficiently defined. The aim of our study was to assess predictors and causes of all-cause hospitalisation during follow-up in patients with DD evaluated using the DICA classification. **Methods:** This prospective observational study included consecutive ambulatory patients with DD evaluated endoscopically in a tertiary gastroenterology centre during a 6-month period. Patients were prospectively followed for 3 years. Demographic and clinical characteristics, DICA classification, treatment, diverticulitis episodes and hospital admissions were recorded. The primary endpoint was any all-cause hospitalisation during follow-up. **Results**: Among 243 patients, all-cause hospitalisation occurred in 101 patients (41.6%), while recurrent hospitalisation occurred in 37 (15.2%). The cumulative hospitalisation burden reached 1607 hospital days. Acute diverticulitis occurred in 15 patients (6.2%), with only one diverticulitis-related hospitalisation, and no diverticular disease-related surgery was recorded. In multivariable analysis, age (OR 1.49; 95% CI 1.06–2.11; *p* = 0.023) and respiratory comorbidity (OR 5.31; 95% CI 1.49–18.88; *p* = 0.010) were independently associated with hospitalisation, whereas DICA ≥ 2 was not. However, the estimate for respiratory comorbidity should be interpreted cautiously because it was based on only 14 affected patients and had a wide confidence interval. **Conclusions**: Although all-cause hospitalisation was common and driven predominantly by age and comorbidity rather than diverticular disease severity, diverticular disease-specific outcomes were generally favourable in this stable ambulatory cohort.

## 1. Introduction

Diverticular disease is one of the most common disorders of the gastrointestinal tract and its prevalence increases markedly with age. Although most individuals with diverticulosis remain asymptomatic, a clinically relevant subgroup develops symptomatic uncomplicated diverticular disease, recurrent abdominal symptoms, acute diverticulitis or complications requiring hospital care [1,2,3]. Consequently, diverticular disease represents an important clinical and healthcare burden, particularly in ageing populations.

Over recent years, increasing attention has been paid not only to acute diverticulitis-related outcomes, but also to the broader impact of diverticular disease on healthcare utilisation [4,5]. Repeated hospitalisations, prolonged hospital stays, recurrent diagnostic procedures and chronic symptom burden may contribute substantially to healthcare costs and reduced quality of life. Whether diverticular disease itself remains a major determinant of long-term clinical outcomes after diagnosis, however, is much less clear.

The Diverticular Inflammation and Complication Assessment (DICA) classification was developed as a structured endoscopic tool enabling objective stratification of diverticular disease severity [6]. Previous studies by Tursi et al. demonstrated that DICA may predict disease-specific outcomes, particularly acute diverticulitis and the need for surgery [7]. However, whether endoscopic severity also predicts broader long-term clinical outcomes beyond diverticular disease-specific events remains insufficiently defined.

This distinction is clinically relevant because patients with diverticular disease are often older and frequently have multiple comorbidities [8,9]. Therefore, long-term healthcare utilisation may reflect general clinical vulnerability and multimorbidity rather than diverticular disease progression itself. As most previous studies have evaluated disease-specific outcomes, whether these adequately reflect the overall long-term clinical course of patients after diagnosis remains uncertain.

The aim of the present study was to evaluate the frequency, causes and predictors of all-cause hospitalisation during 3-year follow-up in a Polish real-world cohort of patients with diverticular disease evaluated according to the DICA classification.

## 2. Materials and Methods

### 2.1. Study Design and Population

This prospective observational cohort study included consecutive patients referred for outpatient colonoscopy for the diagnostic evaluation of gastrointestinal or extraintestinal conditions, in whom diverticular disease was confirmed endoscopically. Patients were enrolled at a tertiary gastroenterology centre during a 6-month recruitment period (1 February 2017 to 31 July 2017) and were subsequently followed prospectively for 3 years after baseline assessment.

The indication for colonoscopy included colorectal cancer surveillance, surveillance after polypectomy, scheduled polypectomy, abdominal pain, gastrointestinal bleeding, verification of computed tomography (CT) findings, mixed bowel habits, diarrhoea, anaemia, constipation, cancer of unknown primary, weight loss, preventive colonoscopy outside the organised screening programme. In the ambulatory pathway at the study centre, appointments were scheduled administratively on the basis of the referral, without a separate specialist qualification visit before the day of colonoscopy. Therefore, when the referral did not clearly state the indication, it was not retrospectively inferred from the clinical history and was classified as “not clearly documented in the referral”.

Inclusion criteria comprised age ≥ 18 years and endoscopic diagnosis of diverticulosis. Patients with inflammatory bowel disease, ischaemic colitis, previous colonic resection, severe hepatic or renal failure, active malignancy, pregnancy, radiological features of acute diverticulitis at baseline, or inability to provide informed consent were excluded.

Baseline demographic, clinical and endoscopic data, including DICA classification, symptoms, comorbidities and treatment, were recorded in a dedicated database. Endoscopic examinations were performed by experienced endoscopists trained in the use of the DICA classification before study initiation.

Follow-up data, including information on disease course, diverticular disease symptoms, treatment, diverticulitis episodes confirmed by healthcare professionals, complications and hospital admissions, were obtained during outpatient visits or scheduled telephone interviews, with an additional review of available medical documentation when necessary. The study was reported in accordance with the STROBE (Strengthening the Reporting of Observational Studies in Epidemiology) statement for cohort studies [10].

### 2.2. Baseline Clinical Variables

Comorbidities were grouped into clinically relevant categories, including cardiovascular disease, respiratory disease, metabolic disease, rheumatic disease, oncological disease, benign prostate hypertrophy requiring pharmacotherapy, thyroid dysfunction, kidney disease and other chronic conditions. Multimorbidity was additionally defined as the presence of at least two comorbidities. Respiratory comorbidity was defined as a chronic respiratory diagnosis recorded in the baseline medical history. This category comprised chronic obstructive pulmonary disease (COPD), asthma, and chronic respiratory failure requiring long-term oxygen therapy (LTOT).

At baseline, abdominal pain, bloating, constipation and diarrhoea were recorded as binary variables. For regression analyses, baseline symptom burden was defined as the sum of bloating, constipation and diarrhoea (range 0–3); abdominal pain was recorded separately and was not included in this composite score. During follow-up, symptom severity was assessed using a 0–10 scale. The binary baseline assessment was used to characterise the presence of predefined symptoms at study entry, whereas the 0–10 scale used during follow-up was intended to capture changes in symptom severity over time. Patients were not prospectively classified as having symptomatic uncomplicated diverticular disease (SUDD) or asymptomatic diverticulosis; therefore, baseline symptom status is reported descriptively rather than as a diagnostic category. A history of previous diverticulitis was recorded from the baseline medical history. Pharmacological treatment was recorded at baseline and during follow-up and included rifaximin, antispasmodics, laxatives, mesalazine, antidiarrhoeal agents and analgesics. For the regression analyses, “scheduled therapy after DICA” was defined as the prescription of any of these treatments.

### 2.3. Outcomes

The primary endpoint was any all-cause hospitalisation during available follow-up. This endpoint was selected to evaluate the overall long-term clinical course of patients with diverticular disease in routine ambulatory care rather than diverticular disease-specific events alone. All-cause hospitalisation was considered a clinically meaningful endpoint because it captures healthcare utilisation arising from the combined effects of disease severity, ageing and comorbidity and therefore represents a patient-centred outcome.

Secondary endpoints included recurrent hospitalisation, cumulative number of hospital days, causes of hospitalisation, acute diverticulitis and diverticular disease-related surgery.

Hospital admissions were classified into the following categories: gastrointestinal causes other than diverticular disease, cardiovascular, infectious, oncological, non-gastrointestinal (non-GI) surgical causes and other. Diverticular disease-related surgery during follow-up was assessed descriptively.

### 2.4. Ethics

All patients provided written informed consent before inclusion in the study. The study protocol conformed to the ethical guidelines of the 1975 Declaration of Helsinki (6th revision, 2008) and was approved by the institutional bioethical committee (41/PB/2016). The analysed cohort originated from a prospective observational study evaluating the clinical utility of the DICA classification. It was registered at ClinicalTrials.gov (NCT02758860). The present report represents a secondary post hoc analysis of this registered cohort; all-cause hospitalisation was not the prespecified primary endpoint, and no a priori sample-size calculation was performed for the present analysis.

### 2.5. Statistical Analysis

Continuous variables were presented as median with interquartile range (IQR). Categorical variables were presented as counts and percentages.

Comparisons between patients with and without hospitalisation were performed using the Mann–Whitney U test for continuous variables and the chi-square test or Fisher’s exact test for categorical variables, as appropriate.

The primary endpoint was analysed using univariable logistic regression. Variables considered clinically relevant were then included in multivariable logistic regression models. Candidate variables included age, sex, body mass index (BMI), multimorbidity, respiratory comorbidity, DICA classification, baseline symptom burden and scheduled therapy after DICA. Variables were selected on clinical grounds to represent demographic characteristics, overall comorbidity, disease-specific severity, baseline symptom burden and treatment exposure, rather than solely on univariable statistical significance. Model robustness was assessed by calculating the number of outcome events per fitted predictor and by evaluating multicollinearity using variance inflation factors (VIFs). Because respiratory disease contributed to the definition of multimorbidity, sensitivity analyses were performed by refitting the model after excluding either multimorbidity or respiratory disease.

As a supplementary time-to-event analysis, time to first all-cause hospitalisation was analysed using Kaplan–Meier curves and Cox proportional hazards regression. Because follow-up data were collected at yearly intervals, time to first hospitalisation was defined as the first follow-up year in which hospitalisation was recorded. Patients without hospitalisation were censored at the last available follow-up assessment. DICA was analysed both descriptively as a three-level classification and in regression models as DICA 1 versus DICA ≥ 2. Logistic regression results were expressed as odds ratios (OR), while Cox regression results were expressed as hazard ratios (HR), both with 95% confidence intervals (CI). A two-sided *p* value < 0.05 was considered statistically significant. Tied event times were handled using Efron’s method.

In an additional sensitivity analysis, death before the first documented all-cause hospitalisation was treated as a competing event, and the cumulative incidence function for hospitalisation was estimated using the Aalen–Johansen method. Because hospitalisations were recorded at yearly follow-up assessments, their occurrence was assigned to months 12, 24 or 36, whereas time to death was estimated in months from information obtained during follow-up. Patients who experienced a documented hospitalisation before death were classified as having reached the hospitalisation endpoint.

Statistical analyses were performed using Statistica version 14.1.0 (TIBCO Software Inc., Palo Alto, CA, USA) and Python version 3.13.5 with the statsmodels package version 0.14.6.

### 2.6. Use of Artificial Intelligence Tools

During the preparation of this manuscript, the authors used ChatGPT (OpenAI, San Francisco, CA, USA) to improve language readability, assist with English spelling and formatting, and support preparation of the graphical abstract. The authors subsequently reviewed and edited the output as needed and take full responsibility for the content of the manuscript.

## 3. Results

### 3.1. Study Population

A total of 275 patients with diverticular disease were included in the cohort. Complete 3-year follow-up was available in 210 patients, incomplete follow-up in 33. Twenty-three patients were lost to follow-up. The main analysis included 243 patients with available follow-up data. Nine patients died during follow-up, at a median of 11 months from baseline (IQR 9–18). Five deaths occurred during the first year, three during the second year and one during the third year. These patients were additionally included in the competing-risk sensitivity analysis.

In the analysed cohort, median age was 65.8 years, and 142 patients were female. Most patients were classified as DICA 1. Comorbidities were common, and the median number of comorbidities was 1 (IQR 0–2). Cardiovascular disease was the most prevalent comorbidity, followed by oncological and metabolic diseases. Among the 14 patients with respiratory comorbidity, 8 had COPD, 5 had asthma, and 1 had chronic respiratory failure requiring LTOT. One patient classified as having asthma additionally had pulmonary calcifications/nodules. Detailed baseline characteristics are presented in Table 1.

Colonoscopy was most commonly performed for surveillance or planned polypectomy (91/243, 37.4%), including colorectal cancer surveillance in 35 patients (14.4%), surveillance after polypectomy in 31 (12.8%), and scheduled polypectomy in 25 (10.3%). Diagnostic symptom- or alarm-related indications accounted for 80 procedures (32.9%), including abdominal pain in 21 patients (8.6%), gastrointestinal bleeding in 17 (7.0%), mixed bowel habits in 11 (4.5%), diarrhoea and anaemia in 10 each (4.1%), constipation in 6 (2.5%), cancer of unknown primary in 4 (1.6%), and weight loss in 1 (0.4%). Verification of CT findings accounted for 12 procedures (4.9%), and preventive colonoscopy outside the organised screening programme for 10 (4.1%). In 50 patients (20.6%), the indication was not clearly documented in the referral. At baseline, 140 of 243 patients (57.6%) had no recorded abdominal pain, bloating, constipation or diarrhoea, whereas 103 patients (42.4%) reported at least one of these symptoms. Abdominal pain was reported by 55 patients (22.6%), bloating by 65 (26.7%), constipation by 73 (30.0%) and diarrhoea by 69 (28.4%). Symptoms were not mutually exclusive. A history of previous diverticulitis was documented in two patients (0.8%).

Pharmacological treatment after DICA was prescribed in 71 of 243 patients (29.2%): antispasmodics in 39 patients, rifaximin in 21, laxatives in 5, mesalazine in 3, antidiarrhoeal agents in 2 and analgesics in 1.

### 3.2. Hospitalisation Burden During Follow-Up

All-cause hospitalisation occurred in 101 patients (41.6%), while recurrent hospitalisation was observed in 37 patients (15.2%). Recurrent admissions accounted for 1071 of 1607 cumulative hospitalisation days (66.6%). The median cumulative length of hospital stay among hospitalised patients was 7 days (IQR 4–14). Causes of hospitalisation were heterogeneous and were predominantly related to cardiovascular, oncological and non-diverticular gastrointestinal conditions (Table 2 and Table 3). Acute diverticulitis occurred in 15 patients (6.2%), including one diverticulitis-related hospitalisation, and no diverticular disease-related surgery was recorded.

### 3.3. Comparison of Patients with and Without Hospitalisation

Patients who were hospitalised during follow-up were older than those without hospitalisation (median age 68.9 vs. 64.4 years; *p* = 0.018). Hospitalised patients also had a higher overall comorbidity burden compared with non-hospitalised patients (*p* = 0.045). Respiratory comorbidity was more frequent among hospitalised than non-hospitalised patients (9.9% vs. 2.8%; *p* = 0.025). There were no significant differences in sex, BMI, smoking status, appendectomy history, cardiovascular disease, metabolic disease, oncological disease, DICA severity or baseline symptom burden. Hospitalisation occurred in 87 of 203 patients with DICA 1, 9 of 29 patients with DICA 2 and 5 of 11 patients with DICA 3. The distribution of hospitalisation did not differ significantly across DICA categories (*p* = 0.465).

### 3.4. Predictors of All-Cause Hospitalisation

In univariable logistic regression, older age was associated with increased odds of hospitalisation (OR 1.44 per 10 years; 95% CI 1.06–1.95; *p* = 0.019). Respiratory comorbidity was also associated with hospitalisation (OR 3.79; 95% CI 1.15–12.45; *p* = 0.028). DICA ≥ 2 was not associated with increased odds of all-cause hospitalisation. Multimorbidity showed a non-significant trend towards higher odds of hospitalisation. Baseline symptom burden was not significantly associated with hospitalisation. In multivariable analysis including age, sex, BMI, multimorbidity, respiratory comorbidity, DICA ≥ 2, baseline symptom burden and scheduled therapy after DICA, only age and respiratory comorbidity remained independently associated with hospitalisation. The adjusted OR for age was 1.49 per 10 years (95% CI 1.06–2.11; *p* = 0.023), and the adjusted OR for respiratory comorbidity was 5.31 (95% CI 1.49–18.88; *p* = 0.010). DICA ≥ 2 remained non-significant (adjusted OR 0.61; 95% CI 0.29–1.29; *p* = 0.194). The multivariable model included 243 patients, 101 hospitalisation events and eight predictors, corresponding to 12.6 events per predictor. All VIF values were low (maximum 1.30), providing no evidence of relevant multicollinearity. After exclusion of multimorbidity, the estimate for respiratory disease remained essentially unchanged (OR 5.23; 95% CI 1.51–18.10; *p* = 0.009), whereas exclusion of respiratory disease did not reveal an association of either multimorbidity or DICA with hospitalisation. These sensitivity analyses supported the stability of the main findings. The primary multivariable model is presented in Table 4, while detailed sensitivity and univariable analyses are provided in Tables S1 and S2, respectively.

### 3.5. Recurrent Hospitalisation

In univariable logistic regression, no baseline variable was significantly associated with recurrent hospitalisation. DICA ≥ 2 was not associated with recurrent hospitalisation (OR 0.40; 95% CI 0.12–1.38; *p* = 0.149). Scheduled therapy after DICA was not associated with recurrent hospitalisation (OR 1.03; 95% CI 0.48–2.22; *p* = 0.941). In multivariable logistic regression, no independent predictor of recurrent hospitalisation was identified. DICA ≥ 2 remained non-significant (adjusted OR 0.39; 95% CI 0.11–1.34; *p* = 0.134). Scheduled therapy after DICA was also not associated with recurrent hospitalisation (adjusted OR 1.08; 95% CI 0.48–2.42; *p* = 0.847).

### 3.6. Time to First Hospitalisation

**Figure 1:**
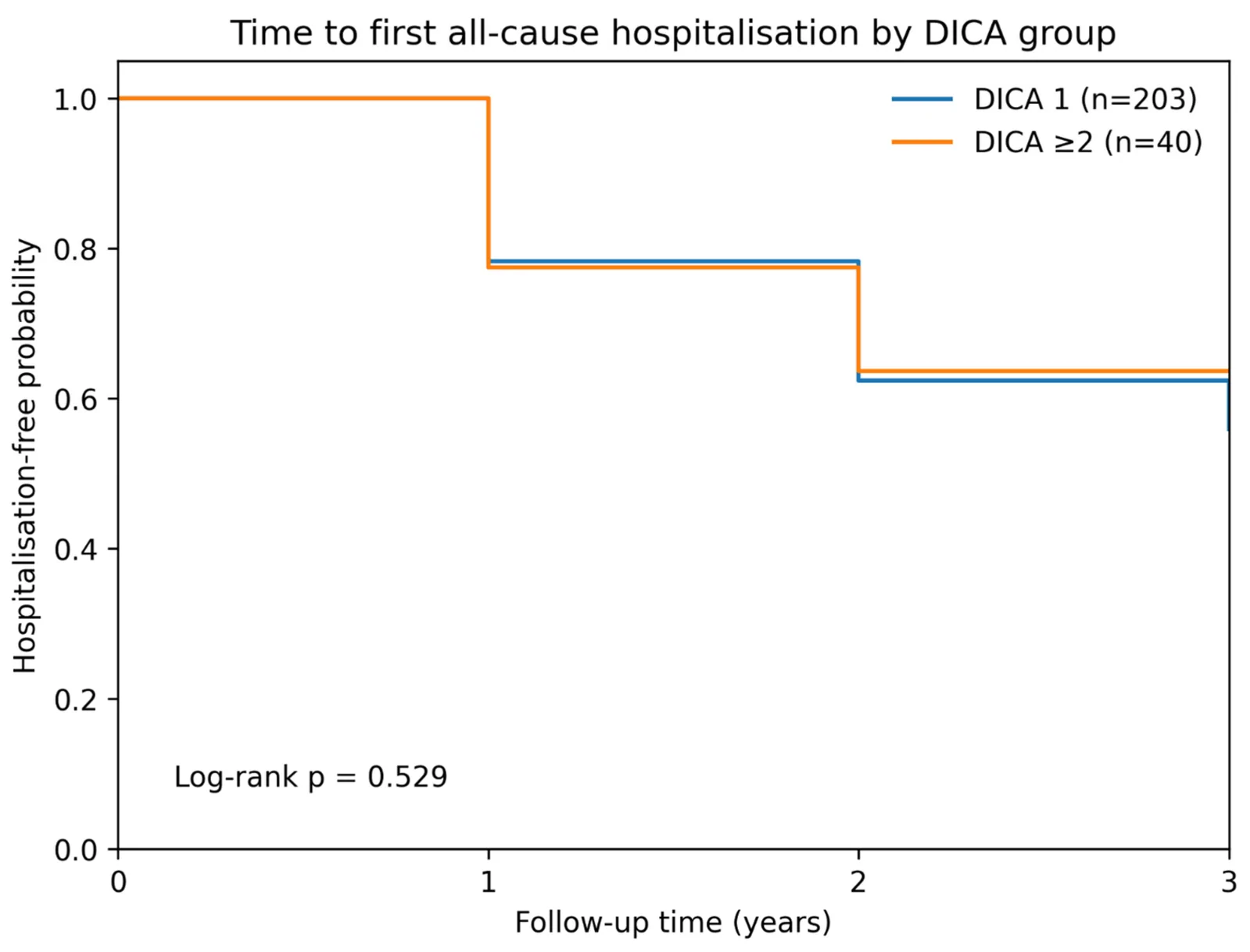
Kaplan–Meier curves for all-cause hospitalisation-free survival according to baseline DICA classification (DICA 1 vs. DICA ≥ 2). No significant difference in hospitalisation-free survival was observed between patients with DICA 1 and DICA ≥ 2 (log-rank *p* = 0.529).

In the supplementary time-to-event analysis, 101 first hospitalisation events were recorded among 243 patients. Fifty-three events occurred during the first year, 36 during the second year and 12 during the third year of follow-up. Kaplan–Meier analysis showed no significant association between DICA severity and all-cause hospitalisation-free survival during follow-up (Figure 1).

In univariable Cox regression, older age and respiratory comorbidity were associated with shorter time to first hospitalisation, whereas DICA ≥ 2 was not (Table S3). In multivariable Cox regression, both older age (adjusted HR 1.33 per 10 years; 95% CI 1.03–1.71; *p* = 0.027) and respiratory comorbidity (adjusted HR 3.00; 95% CI 1.50–6.02; *p* = 0.002) remained independently associated with shorter time to hospitalisation. DICA ≥ 2 remained non-significant (adjusted HR 0.80; 95% CI 0.45–1.43; *p* = 0.459).

In the competing-risk sensitivity analysis including 252 patients, one of the nine deceased patients had experienced a documented hospitalisation before death, while the remaining eight deaths were treated as competing events. The estimated 3-year cumulative incidence of first all-cause hospitalisation was 41.8% (95% CI 35.6–48.0%). The corresponding cumulative incidence was 42.6% in patients with DICA 1 and 37.0% in those with DICA ≥ 2, indicating that accounting for death as a competing event did not materially alter the principal findings.

## 4. Discussion

While diverticulitis is recognised as an important cause of gastrointestinal hospitalisation and healthcare expenditure worldwide, previous studies have focused predominantly on diverticular disease-related admissions, surgery, recurrence and administrative epidemiological outcomes rather than all-cause hospitalisation burden in ambulatory patients with diverticular disease [5,11,12,13,14]. Most available data originate from retrospective analyses of large national databases or studies evaluating acute diverticulitis requiring hospitalisation [11,12,13].

The present study addressed a clinically important question: whether, after endoscopic diagnosis of diverticular disease, the disease itself continues to determine patients’ long-term clinical course or whether subsequent outcomes are driven predominantly by ageing and accompanying chronic diseases. Using all-cause hospitalisation as a patient-centred measure of long-term clinical trajectory, we found that hospitalisation during follow-up was frequent, affecting more than 40% of patients. However, hospitalisation risk was not associated with endoscopic severity assessed by the DICA classification. Instead, older age and respiratory comorbidity emerged as the principal predictors of hospitalisation. Importantly, recurrent hospitalisations, although observed in a minority of patients, generated approximately two-thirds of cumulative hospital days. The distribution of hospitalisation causes in our cohort appeared broadly consistent with contemporary European inpatient statistics, in which cardiovascular diseases represent one of the leading causes of hospital discharge, followed by oncological and gastrointestinal conditions [15]. Moreover, the median cumulative length of hospital stay observed in our cohort was comparable to contemporary European and Polish estimates for hospitalised older adults [16]. These observations suggest that, in ambulatory patients with diverticular disease, the overall pattern of hospitalisation may reflect ageing, multimorbidity and general clinical vulnerability rather than diverticular disease-specific complications alone. Despite frequent hospitalisations, very few were directly attributable to diverticular disease itself. In our cohort, diverticular disease appeared to coexist with, rather than dominate, the broader clinical course of ageing patients. In practical terms, diverticular disease itself contributed only modestly to long-term hospitalisation burden once patients entered ambulatory follow-up. This observation also illustrates why all-cause rather than disease-specific hospitalisation provides a more comprehensive assessment of long-term outcomes, as disease-specific endpoints alone may overestimate the long-term clinical impact of diverticular disease by ignoring the competing influence of ageing and multimorbidity. Conversely, because all-cause hospitalisation is strongly influenced by ageing and unrelated comorbidities, the use of this broad endpoint may have diluted an association between DICA severity and diverticular disease-specific outcomes.

This finding is clinically important because DICA was developed and validated primarily as a disease-specific tool for assessing endoscopic severity and predicting diverticular disease-related outcomes, including acute diverticulitis recurrence and surgery [4,6,7]. Our findings therefore do not contradict previous DICA studies published by Tursi et al., but rather extend them by demonstrating that endoscopic severity may not necessarily reflect broader healthcare utilisation or overall clinical frailty. The lack of association between DICA severity and all-cause hospitalisation should therefore be interpreted as reflecting the fundamentally different nature of the analysed endpoint rather than limited utility of the DICA classification itself. However, because only 40 patients were classified as DICA ≥ 2, the absence of a statistically significant association should be interpreted cautiously, as the study may have had limited power to detect a modest effect of higher DICA severity on all-cause hospitalisation. Indeed, had diverticular disease remained the principal determinant of subsequent healthcare utilisation, one would expect increasing DICA severity to translate into a higher overall hospitalisation risk despite competing causes of admission. The heterogeneous causes of hospitalisation observed in this study further support this interpretation. Gastrointestinal causes other than diverticular disease represented only a minority of admissions, while cardiovascular, oncological, infectious and non-gastrointestinal surgical causes together accounted for most hospitalisation episodes. Notably, only a single hospitalisation was directly related to acute diverticulitis despite the prospective follow-up over 3 years. These findings suggest that, in stable ambulatory patients without acute diverticulitis at baseline, the clinical course of diverticular disease is generally favourable, while long-term healthcare utilisation is determined predominantly by accompanying chronic diseases rather than diverticular disease progression. Our findings may also be viewed in the context of recent population-based observations published by Cameron and colleagues, suggesting that uncomplicated diverticular disease itself may not necessarily confer an adverse long-term prognosis once major confounding factors are taken into account. In a large United Kingdom primary care cohort, Cameron et al. demonstrated that after adjustment for age, sex and comorbidities, uncomplicated diverticular disease was not associated with increased mortality, whereas complicated diverticulitis remained associated with excess risk. The authors proposed that the apparent adverse prognosis observed in unadjusted analyses may largely reflect ageing and multimorbidity rather than diverticular disease itself. Although our study evaluated hospitalisation burden rather than mortality, the present findings appear broadly consistent with this concept [17]. Together, these findings reinforce the concept that uncomplicated diverticular disease should not be regarded as a condition associated with an intrinsically poor long-term prognosis. Our results complement these observations by demonstrating that, once broader patient-centred outcomes are considered, ageing and multimorbidity appear to outweigh diverticular disease severity in determining subsequent healthcare utilisation.

An additional important finding was the disproportionate contribution of recurrent hospitalisations to overall healthcare burden. Although recurrent admissions occurred in only 15.2% of patients, they accounted for approximately two-thirds of all cumulative hospitalisation days. This observation suggests that a relatively small subgroup of clinically vulnerable patients may generate a substantial proportion of healthcare utilisation. Similar patterns of recurrent disease course and long-term healthcare impact have been reported in studies evaluating recurrent diverticulitis and long-term outcomes after conservative management [18]. However, unlike previous studies focused primarily on diverticulitis recurrence, our findings demonstrate that recurrent all-cause hospitalisations constitute an important component of the overall clinical burden in ambulatory patients with diverticular disease.

The association between respiratory comorbidity and hospitalisation is also noteworthy. Although respiratory diseases were present only in a relatively small subgroup of patients, they were associated with a markedly increased risk of hospitalisation. This finding may reflect the broader impact of frailty, reduced physiological reserve and multimorbidity on healthcare utilisation in ageing populations. Similar observations have been reported in geriatric population studies, such as the nationwide PolSenior2 study, in which multimorbidity and chronic systemic diseases substantially increased hospitalisation risk and healthcare burden [16,19].

Our findings may have practical clinical implications. The management of patients with diverticular disease should combine disease-specific risk stratification with a broader assessment of comorbidity and healthcare vulnerability. In older patients, especially those with multimorbidity or chronic respiratory disease, the risk of hospitalisation may be determined more by systemic clinical factors than by diverticular disease severity itself. Equally important, clinicians may reassure many patients that diverticular disease itself is unlikely to determine their long-term prognosis once serious complications have been excluded. For many patients, this information is clinically important because it may help reduce unnecessary concerns that diverticular disease will dominate their future health and quality of life.

The study has some limitations. First, this was a single-centre, single-country prospective observational cohort, which may limit the generalisability of the findings to other populations and healthcare systems. The findings have not been externally validated. In addition, this was a secondary post hoc analysis, and no a priori sample-size calculation was performed for the all-cause hospitalisation endpoint; consequently, the study may have had limited power to detect modest associations. Second, only 11 patients (4.5%) had DICA 3 disease, limiting the statistical power to evaluate outcomes in patients with severe endoscopic disease. Accordingly, the present findings should be interpreted as applying primarily to stable ambulatory patients without acute diverticulitis at baseline and predominantly mild endoscopic disease, rather than to patients with acute, complicated or advanced diverticular disease. Third, respiratory comorbidity was present in a relatively small and clinically heterogeneous subgroup of patients and therefore the strong association observed in multivariable analysis should be interpreted cautiously and confirmed in larger cohorts. Fourth, in 20.6% of patients, the indication for colonoscopy could not be reliably classified from the referral documentation. This should not be interpreted as absence of a clinical indication; rather, it reflects the referral-based ambulatory pathway at the study centre, in which procedures were scheduled without a separate pre-procedure specialist qualification visit. This limits detailed characterisation of referral indications but does not affect ascertainment of baseline endoscopic findings or prospectively collected follow-up outcomes. Because hospitalisations were recorded only at annual follow-up assessments, the Kaplan–Meier and Cox analyses had limited temporal resolution and should therefore be regarded as supplementary. Finally, exact dates of death were not available, and time to death was reconstructed to the nearest month from information obtained during follow-up. Nevertheless, only nine deaths occurred, and a competing-risk sensitivity analysis produced results that were essentially consistent with the primary analysis.

The strengths of the study include prospective longitudinal follow-up, structured baseline endoscopic assessment using the DICA classification, detailed categorisation of hospitalisation causes and real-world ambulatory cohort design. The broad range of surveillance, therapeutic and diagnostic referral indications further supports the real-world character of the cohort. To the best of our knowledge, this is one of the first prospective studies evaluating all-cause hospitalisation burden and recurrent hospitalisations in ambulatory patients with diverticular disease assessed using the DICA classification.

## 5. Conclusions

Our findings suggest that, in stable ambulatory patients with predominantly mild diverticular disease, long-term outcomes are driven predominantly by ageing and comorbidity rather than diverticular disease itself. Consequently, diverticular disease should be viewed primarily as a chronic gastrointestinal condition that coexists with, rather than dominates, the overall health trajectory of older patients. These findings should not be extrapolated to patients with acute diverticulitis, complicated disease or severe DICA 3 disease.

## Figures and Tables

**Table 1:** Baseline characteristics of patients according to hospitalisation status.

Variable	Total (*n* = 243)	Not Hospitalised (*n* = 142)	Hospitalised (*n* = 101)	*p* Value
Age, years, median (IQR)	65.8 (61.8–74.4)	64.4 (61.6–73.2)	68.9 (62.3–77.0)	**0.018**
Female sex, *n* (%)	142 (58.4)	88 (62.0)	54 (53.5)	0.185
BMI, kg/m^2^, median (IQR)	27.2 (24.4–30.7)	27.4 (24.3–30.4)	26.9 (24.6–30.8)	0.949
Current smoking, *n* (%)	41 (16.9)	21 (14.8)	20 (19.8)	0.304
Former smoking, *n* (%)	84 (34.6)	48 (33.8)	36 (35.6)	0.766
Previous appendectomy, *n* (%)	40 (16.5)	26 (18.3)	14 (13.9)	0.357
Number of comorbidities, median (IQR)	1 (0–2)	1 (0–2)	1 (1–2)	**0.045**
Cardiovascular disease, *n* (%)	127 (52.3)	72 (50.7)	55 (54.5)	0.564
Respiratory disease, *n* (%)	14 (5.8)	4 (2.8)	10 (9.9)	**0.025**
Metabolic disease, *n* (%)	38 (15.6)	20 (14.1)	18 (17.8)	0.429
Rheumatic disease, *n* (%)	19 (7.8)	8 (5.6)	11 (10.9)	0.132
Oncological disease, *n* (%)	40 (16.5)	23 (16.2)	17 (16.8)	0.895
DICA 1, *n* (%)	203 (83.5)	116 (81.7)	87 (86.1)	0.465 *
DICA 2, *n* (%)	29 (11.9)	20 (14.1)	9 (8.9)	
DICA 3, *n* (%)	11 (4.5)	6 (4.2)	5 (5.0)	
Scheduled therapy after DICA, *n* (%)	71 (29.2)	41 (28.9)	30 (29.7)	0.889

DICA—Diverticular Inflammation and Complication Assessment. * Comparisons were performed across DICA 1, DICA 2 and DICA 3 categories. Statistically significant *p* values are shown in bold.

**Table 2:** Hospitalisation outcomes and causes during follow-up.

Outcome	Result
Patients with available follow-up data	243
Any all-cause hospitalisation	101 (41.6%)
Recurrent hospitalisation	37 (15.2%)
Total hospitalisation episodes	144
Total hospital days	1607
Cumulative length of stay among hospitalised patients, median (IQR)	7 (4–14)
Acute diverticulitis during follow-up	15 (6.2%)
Diverticular disease-related surgery	0

**Table 3:** Causes of hospitalisation episodes are presented in the following.

Cause Group	Episodes, *n* (%)	Patients, *n*
Cardiovascular	30 (20.8%)	28
Other	28 (19.4%)	25
Gastrointestinal other than DD	28 (19.4%)	25
Non-GI surgical	25 (17.4%)	23
Oncological	23 (16.0%)	22
Infection	10 (6.9%)	9

DD—diverticular disease; patients may be represented in more than one cause group.

**Table 4:** Multivariable predictors of all-cause hospitalisation.

Variable	Adjusted OR	95% CI	*p* Value
Age, per 10 years	1.49	1.06–2.11	**0.023**
Female sex	0.74	0.43–1.29	0.293
BMI, per 5 kg/m^2^	1.04	0.80–1.35	0.783
Multimorbidity (≥2 comorbidities)	0.97	0.52–1.78	0.912
Respiratory disease	5.31	1.49–18.88	**0.010**
DICA ≥ 2	0.61	0.29–1.29	0.194
Baseline symptom burden	0.87	0.67–1.13	0.304
Scheduled therapy after DICA	1.21	0.66–2.21	0.540

Statistically significant *p* values are shown in bold.

## Data Availability

The data supporting the findings of this study are available from the study guarantor upon reasonable request.

## Supplementary Materials

The following supporting information can be downloaded at: https://www.mdpi.com/article/10.3390/jcm15166385/s1, Table S1: Sensitivity analyses of the multivariable logistic regression model for all-cause hospitalisation; Table S2: Univariable predictors of all-cause hospitalisation; Table S3: Cox proportional hazards analysis for time to first all-cause hospitalisation.

## Abbreviations

The following abbreviations are used in this manuscript:

BMI	Body mass index
COPD	Chronic obstructive pulmonary disease
CT	Computed tomography
DD	Diverticular disease
DICA	Diverticular Inflammation and Complication Assessment
GI	Gastrointestinal
IQR	Interquartile range
LTOT	Long-term oxygen therapy
STROBE	Strengthening the Reporting of Observational Studies in Epidemiology
SUDD	Symptomatic uncomplicated diverticular disease
VIF	Variance inflation factor
