# Supplementary material for: All-Cause Hospitalisation After Endoscopic Diagnosis of Diverticular Disease: A Prospective Real-World Cohort Study
Source: J Clin Med. 2026 Aug 18;15(16):6385. doi: 10.3390/jcm15166385 (PMC13514152; doi:10.3390/jcm15166385)
Supplement: Supplementary file 1 [file jcm-15-06385-s001.zip › jcm-4466700-supplementary.pdf]

Supplementary Materials

Table S1. Sensitivity analyses of the multivariable logistic regression model for all-cause hospitalisation.

Variable	Primary model OR (95% CI)	p value	Model excluding multimorbidity OR (95% CI)	p value	Model excluding respiratory disease OR (95% CI)	p value
Age, per 10 years	1.49 (1.06–2.11)	**0.023**	1.48 (1.07–2.06)	**0.018**	1.34 (0.96–1.87)	0.082
Female sex	0.74 (0.43–1.29)	0.293	0.74 (0.43–1.29)	0.290	0.78 (0.45–1.34)	0.364
BMI, per 5 kg/m²	1.04 (0.80–1.35)	0.783	1.03 (0.80–1.34)	0.796	1.00 (0.77–1.29)	0.971
Multimorbidity (≥2 comorbidities)	0.97 (0.52–1.78)	0.912	—	—	1.15 (0.63–2.07)	0.650
Respiratory disease	5.31 (1.49–18.88)	**0.010**	5.23 (1.51–18.10)	**0.009**	—	—
DICA ≥2	0.61 (0.29–1.29)	0.194	0.61 (0.29–1.29)	0.196	0.66 (0.32–1.36)	0.260
Baseline symptom burden	0.87 (0.67–1.13)	0.304	0.88 (0.68–1.13)	0.302	0.87 (0.68–1.13)	0.299
Scheduled therapy after DICA assessment	1.21 (0.66–2.21)	0.540	1.21 (0.66–2.20)	0.543	1.19 (0.66–2.16)	0.569

The primary analysis included 243 patients and 101 hospitalisation events, corresponding to 12.6 events per predictor. The maximum variance inflation factor in the primary model was 1.30.

Table S2. Univariable predictors of all-cause hospitalisation.

Variable	OR	95% CI	p value
Age, per 10 years	1.44	1.06–1.95	**0.019**
Female sex	0.71	0.42–1.18	0.185
BMI, per 5 kg/m²	0.99	0.77–1.25	0.904
Current smoking	1.42	0.73–2.79	0.305
Former smoking	1.08	0.63–1.85	0.766
Multimorbidity (≥2 comorbidities)	1.51	0.89–2.56	0.123
Cardiovascular disease	1.16	0.70–1.94	0.564
Respiratory disease	3.79	1.15–12.45	**0.028**
Metabolic disease	1.32	0.66–2.65	0.430
Oncological disease	1.05	0.53–2.08	0.895
DICA ≥2	0.72	0.35–1.46	0.358
Baseline symptom burden	0.81	0.65–1.02	0.073
Scheduled therapy after DICA assessment	1.04	0.59–1.82	0.889

Table S3. Cox proportional hazards analysis for time to first all-cause hospitalisation.

Variable	Univariable HR	95% CI	p value	Adjusted HR	95% CI	p value
Age, per 10 years	1.30	1.04–1.63	**0.021**	1.33	1.03–1.71	0.027
Female sex	0.76	0.51–1.12	0.164	0.78	0.52–1.18	0.248
BMI, per 5 kg/m²	0.99	0.83–1.19	0.917	1.02	0.84–1.25	0.847
Multimorbidity (≥2 comorbidities)	1.42	0.96–2.11	0.078	1.07	0.69–1.66	0.762
Respiratory disease	2.34	1.22–4.50	**0.011**	3.00	1.50–6.02	**0.002**
DICA ≥2	0.85	0.48–1.48	0.554	0.80	0.45–1.43	0.459
Baseline symptom burden	0.85	0.71–1.02	0.086	0.90	0.73–1.09	0.281
Scheduled therapy after DICA assessment	1.06	0.69–1.62	0.790	1.23	0.78–1.95	0.369
